# Myoepithelial cells from pleomorphic adenoma are not influenced by tumor conditioned media from breast ductal adenocarcinoma and melanoma cells: An *in vitro* study

**DOI:** 10.3892/ol.2014.2624

**Published:** 2014-10-17

**Authors:** ELIZABETH FERREIRA MARTINEZ, ANA PAULA DIAS DEMASI, MARCELO HENRIQUE NAPIMOGA, CAROLINA AMÁLIA BARCELLOS SILVA, NATALIA FESTUGATTO NAVARINI, NEY SOARES ARAÚJO, VERA CAVALCANTI DE ARAÚJO

**Affiliations:** Department of Oral Pathology, São Leopoldo Mandic Institute and Research Center, Campinas, São Paulo 13045-610, Brazil

**Keywords:** myoepithelial cells, tumor microenvironment, malignant cell lines, salivary gland tumors

## Abstract

Myoepithelial cells have been implicated in the regulation of the transition from *in situ* to invasive neoplasia in salivary gland tumors. Considering the importance of the microenvironment of the tumor, the present *in vitro* study therefore analyzed the morphological and phenotypic changes undergone by benign myoepithelial cells from pleomorphic adenoma (PA) stimulated by tumor-conditioned medium. The benign myoepithelial cells were obtained from PA and were cultured with fibronectin extracellular matrix protein, supplemented with tumor-conditioned medium, which was harvested from breast ductal adenocarcinoma AU-565 and melanoma Hs 852.T cells. The morphological alterations were assessed by immunofluorescence analysis using vimentin antibody. The α-smooth muscle actin (α-SMA) and fibroblast growth factor (FGF)-2 proteins were analyzed by indirect immunofluorescence and quantitative polymerase chain reaction (qPCR). No morphological changes were observed in the myoepithelial cells cultured in fibronectin protein under stimulation from either tumor-conditioned medium. The immunofluorescence results, which were supported by qPCR analysis, revealed that only α-SMA was upregulated in the fibronectin substratum, with or without tumor-conditioned medium obtained from breast ductal adenocarcinoma and melanoma cells. No significant difference in FGF-2 mRNA expression was detected when the cells were cultured either in the tumor-conditioned medium or in the fibronectin substratum. The tumor-conditioned medium harvested from breast ductal adenocarcinoma and melanoma did not affect myoepithelial cell differentiation and function, which was reflected by the fact that there was no observed increase in α-SMA and FGF-2 expression, respectively.

## Introduction

The interactions between stromal cells and tumor cells are important aspects of tumor growth and invasion. In salivary gland tumors, myoepithelial cells have been implicated in the regulation of the transition from *in situ* to invasive neoplasia (1).

Myoepithelial cells exert inhibitory effects on numerous neoplastic phenotypes, including tumor cell growth, invasion and angiogenesis, and have been described as natural tumor suppressors (2–5). Therefore, extracellular matrix-cell interactions are essential not only for normal development, but also for their role in tumorigenesis (6).

*In vivo* modification of the phenotype of benign myoepithelial cells in *in situ* areas of carcinoma ex pleomorphic adenoma (PA) induced by malignant transformation of epithelial cells has been demonstrated, revealing crosstalk between the myoepithelial and adenoma cells (7,8). Due to these studies, an *in vitro* model was used to investigate the role of myoepithelial cells and the tumor microenvironment in salivary gland neoplasms (9). The focus was the influence of extracellular matrix proteins, including basement membrane matrix, type I collagen and fibronectin, on the morphology and differentiation of benign myoepithelial cells from PA that were cultured with medium obtained from the culture of squamous cell carcinoma tumor cells (10). This demonstrated that the extracellular matrix plays an important role in the morphology of benign myoepithelial cells under the influence of squamous cell carcinoma tumor medium, and also plays a role in inducing an increase in the expression of fibroblast growth factor (FGF)-2 and α-smooth muscle actin (α-SMA) in these cells, particularly in the fibronectin substratum.

Considering the interaction between squamous cell carcinoma and myoepithelial cells under the influence of the tumor microenvironment (10), the present *in vitro* study aimed to examine the role of tumor-conditioned medium, obtained from melanoma and breast ductal adenocarcinoma cells, in the morphological and phenotypic alterations of neoplastic benign myoepithelial cells obtained from PA under a fibronectin substratum.

## Materials and methods

### Cell culture

Benign myoepithelial cells were obtained from explants of PA tumors from three different donors, according to the methodology described in previous studies (8–10). The present study was approved by the Ethics Committee of São Leopoldo Mandic Institute and Dental Research Center (Campinas, Brazil; Protocol 09/0014). All patients provided written informed consent.

The cells were cultured in Dulbecco’s modified Eagle’s medium (DMEM; Sigma-Aldrich, St. Louis, MO, USA) supplemented with 1% antimycotic-antibiotic solution (10,000 units penicillin, 10 mg streptomycin and 25 μg/ml amphotericin B in 0.9% sodium chloride; Sigma-Aldrich), supplemented with 10% donor calf serum (Gibco Life Technologies, Carlsbad, CA, USA). The cells were then plated in 60-mm diameter plastic culture dishes and incubated under the standard cell culture conditions of 37°C, 100% humidity, 95% air and 5% CO_2_. Subsequent to reaching confluence, the cells were detached using 0.05% trypsin and subcultured at a density of 110 cells/mm^2^ in 20 μg/ml of fibronectin substratum (Sigma-Aldrich). The cells were then placed in the polystyrene plate or on 13-mm coverslips for the subsequent experiments. The plated benign myoepithelial cells were cultured in DMEM for 24 h prior to being supplemented with tumor-conditioned medium, according to the methodology described by Martinez *et al* (9).

For the *in vitro* induction with tumor-conditioned medium, melanoma Hs 852.T and breast ductal adenocarcinoma AU-565 cells were obtained from the American Type Culture Collection (Manassas, VA, USA). The cell medium was changed 48 h prior to use. Benign myoepithelial cells cultured in DMEM for 24 h were then incubated for four days with the tumor-conditioned medium, which was previously filtered using a 0.22 μm sterile syringe filter (Corning, Inc., Corning, New York, NY, USA). The analysis was also carried out using non-conditioned DMEM as a control.

### Immunofluorescence

The cells grown on coverslips in various substrata were fixed in methanol for 6 min at −20°C, rinsed with phosphate-buffered saline (PBS) and then blocked with 1% bovine albumin in PBS for 30 min at room temperature. The primary antibodies used were FGF-2 (1:50; polyclonal rabbit anti-human; Santa Cruz Biotechnology, Inc., Dallas, TX, USA) and α-SMA (1:50; monoclonal mouse anti-human; Dako, Glostrup, Denmark). The control staining reaction was performed using PBS in place of the primary antibody. The secondary antibody used was biotinylated goat anti-rabbit polyclonal immunoglobulin G (IgG) or goat anti-mouse polyclonal IgG (Vector Laboratories, Inc., Burlingame, CA, USA). Streptavidin, fluorescein-conjugated (Vector Laboratories, Inc.) was used for the second step. The preparations were washed and mounted using DAPI-associated (4′-6-diamidino-2-phenylindole) Vectashield (Vector Laboratories, Inc.) and assessed on a Zeiss Axioskop 2 conventional fluorescence microscope (Carl Zeiss MicroImaging GmbH, Jena, Germany) equipped with 63X Plan Apochromatic 1.4NA and 100X Plan Apochromatic 1.4NA objectives in standard conditions (Carl Zeiss, Oberköchen, Germany). To verify the morphological changes of benign myoepithelial cells obtained from PA cultured with tumor-conditioned medium, the cells were also immunostained with vimentin (1:400; monoclonal mouse anti-human; Dako).

### Quantitative polymerase chain reaction (qPCR)

Total RNA was extracted from PA myoepithelial cells cultured in various conditions using Tri Reagent (Molecular Research Center, Cincinnati, Ohio, USA). The RNA underwent reverse transcription using the Superscript III First Strand cDNA Synthesis kit (Invitrogen, Carlsbad, CA, USA), according to the manufacturer’s instructions. The primer sets were as follows: α-SMA, forward; 5′-ATGCTCCCAGGGCTGTTTT-3′ and reverse; 5′-GCTTCGTCACCCACGTAGCT-3′; FGF-2, forward, 5′-GTGCTAACCGTTACCTGGCTAT-3′ and reverse; 5′-CCAATCGTTCAAAAAAGAAACAC-3′; and for the internal gene reference β-actin (ACTB), forward; 5′-AGGCCAACCGCGAGAAG-3′ and reverse; 5′-ACAGCC TGGATAGCAACGTACA-3′. qPCR was performed using a 7300 Real Time PCR system (Applied Biosystems, Foster City, CA, USA) with SYBR Green as detection dye. The cycling conditions were 10 min at 95°C followed by 40 cycles of 95°C for 15 sec and 60°C for 1 min. The quantification data were analyzed using the SDS System Software (Applied Biosystems) and the relative expression levels were calculated according to the comparative Ct method, as 2^−ΔΔCt^. Each qPCR experiment was repeated three times.

### Statistics

The results are expressed as the mean ± standard deviation. In order to compare the results between the various conditions, two-way analysis of variance and post hoc Bonferroni test were performed, with a significance level of 0.05.

## Results

### Myoepithelial cell morphology

**Figure 1:**
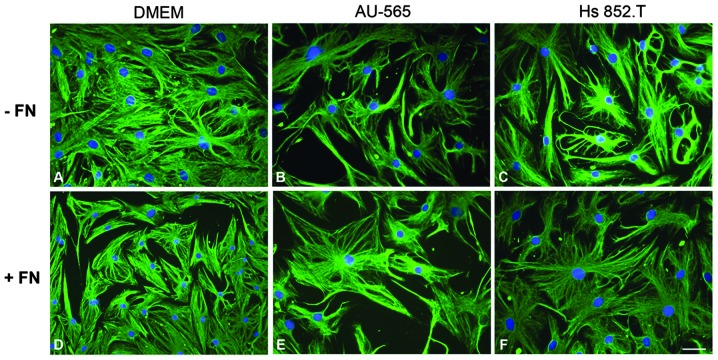
Immunostaining for vimentin in myoepithelial cells from pleomorphic adenoma on (A–C) polystyrene (−FN) and (D–F) fibronectin substratum (+FN). The cells exhibited a stellate morphology independent of the tumor-conditioned medium stimulation. Medium: (A and D) Dulbecco’s modified Eagle’s medium (DMEM); (B and E) breast ductal adenocarcinoma AU-565 cell-conditioned medium; and (C and F) melanoma Hs 852.T cell-conditioned medium. Nuclei stained with DAPI appear in blue. Scale bar, 100 μm. Magnification, ×400.

In order to verify whether the tumor-conditioned media from breast ductal adenocarcinoma AU-565 and melanoma Hs 852.T cells altered myoepithelial cell morphology, the cells were examined using indirect immunofluorescence for vimentin (Fig. 1). There was no change in the morphology of myoepithelial cells plated with fibronectin substratum *in vitro*. In all the studied conditions, the cells exhibited stellate and polyhedral morphology, even when supplemented with the tumor-conditioned media.

### Myoepithelial cell immunophenotype

**Figure 2:**
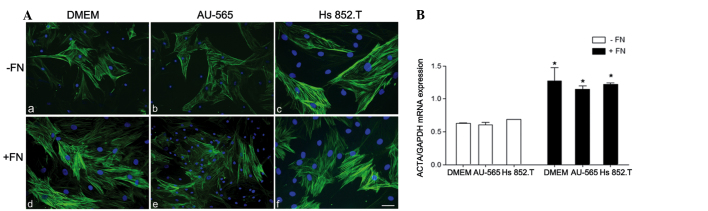
(A) Immunostaining for α-smooth muscle actin (α-SMA) in myoepithelial cells from pleomorphic adenoma on (a–c) polystyrene (−FN) and (d–f) fibronectin substratum (+FN). α-SMA was heterogeneously immunoexpressed in the myoepithelial cells in all the studied conditions. However, in the +FN cells, there was an increase in α-SMA immunostaining. The nuclei stained with DAPI appear in blue. Scale bars: A, B and E, 50 μm; magnification, ×200; and C, D and F, 100 μm; magnification, ×400. (B) Relative α-SMA mRNA expression. The expression of α-SMA was significantly upregulated in all +FN conditions. ^*^+FN vs. −FN, P<0.05. Medium: a and d, Dulbecco’s modified Eagle’s medium (DMEM); b and e, breast ductal adenocarcinoma AU-565 cell-conditioned medium; and c and f, melanoma Hs 852.T cell-conditioned medium.

**Figure 3:**
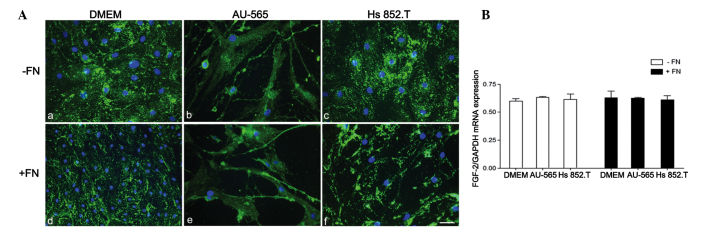
(A) Immunostaining for fibroblast growth factor (FGF)-2 in myoepithelial cells from pleomorphic adenoma on a–c) polystyrene (−FN) and (d–f) fibronectin substratum (+FN). FGF-2 was immunoexpressed as punctuate deposits in the cytoplasm in all the studied conditions. Nuclei stained with DAPI appear in blue. Scale bars: a-c, e and f, 100 μm; magnification ×400; and d, 50 μm; magnification, ×200. (B) Relative FGF-2 mRNA expression. No statistical difference in FGF-2 expression was observed between the studied conditions. ^*^+FN vs. −FN, P<0.05. Medium: a and d, Dulbecco’s modified Eagle’s medium (DMEM); b and e, breast ductal adenocarcinoma AU-565 cell-conditioned medium; and c and f, melanoma Hs 852.T cell-conditioned medium.

The immunophenotype of the myoepithelial cells alone or supplemented with the various tumor-conditioned media was assessed using indirect immunofluorescence supported by qPCR analysis (Figs. 2 and 3).

The results demonstrated that only α-SMA was upregulated in benign PA myoepithelial cells in tumor-conditioned media from breast ductal adenocarcinoma and melanoma cells in the fibronectin substratum (Fig. 2). As previously demonstrated (10), α-SMA was also heterogeneously immunoexpressed in myoepithelial cells. No α-SMA immunophenotypical differences or differences in mRNA expression were observed independent of the studied conditions of tumor-conditioned media stimulation and DMEM.

FGF-2 was immunoexpressed in the myoepithelial cells as punctuate deposits throughout the cytoplasm in all studied conditions (Fig. 3). No difference in FGF-2 mRNA expression was detected when the cells were cultured either in the tumor-conditioned medium or in the fibronectin substratum.

## Discussion

The interaction between cells and the surrounding extracellular matrix is an important component of the development and function of numerous biological events, including normal development and tumorigenesis (11). Although the tumor microenvironment has been extensively studied, including the important role of the extracellular matrix associated with the secretion of numerous molecules and the stromal cells, the process remains unclear. An *in vitro* model that mimics an *in situ* scenario of carcinoma ex PA (CXPA) has been developed (9) in order to investigate the crosstalk between myoepithelial and cancer cells. A squamous cell carcinoma cell line is used as the source of the tumor-conditioned medium based on the CXPA characteristics. In this type of tumor, epithelial cells can undergo malignant change and present features similar to those of a squamous cell carcinoma (12). In the present study, various tumor-conditioned media were used in order to investigate the effects of the secretory factors released by carcinoma cells on myoepithelial cells, which may be important to determine tumor behavior. Two malignant cell lines were studied, the breast ductal adenocarcinoma AU-565 cell line, which exhibits similarities to the salivary gland neoplasm (13,14) and the melanoma Hs 852.T cell line, due to cytokine and growth factor production-associated aggressiveness (15).

The present results revealed that, despite the several growth factors these cell lines secrete and using the proposed *in vitro* model, no alteration in myoepithelial cell morphology was detected. These findings are in line with those of a previous study (10) that used squamous cell carcinoma-conditioned medium, in which the myoepithelial cells exhibited a polyhedral and stellate morphology in the fibronectin substratum, forming sites of adhesion and focal contact with the matrix, a fundamental event for malignant cell colonization. In salivary gland neoplasms, unlike tenascin, fibronectin is not present in the tumor invasion front (16,17). In breast tissue, however, the fibronectin in the stromal extracellular matrix may assist the process of tumorigenesis (18). Furthermore, the extracellular matrix may influence intracellular signaling and cell cycle control, thus contributing to tumor cell migration and invasion (19). Based on the results obtained in a previous study, the morphological aspect of the myoepithelial cells in fibronectin, regardless of the malignant *in situ* condition, appeared to have impaired their tumor suppressor function.

The present data revealed that the expression of α-SMA was upregulated in benign myoepithelial cells from PA in fibronectin. This finding corroborates those from a previous study (10), thus highlighting the importance of this extracellular matrix protein in triggering cell-matrix interactions and subsequent changes in actin cytoskeleton, which are essential for the control of directional cell migration and invasion (11).

Notably, no difference was observed between the morphology of the studied groups, indicating that the tumor-conditioned media from breast ductal adenocarcinoma and melanoma cells did not influence myoepithelial cell differentiation. de Araújo *et al* (7) demonstrated that the myoepithelial cells surrounding regions of malignant transformation were phenotypically different from the benign myoepithelial cells of the PA. In addition, the former expressed a higher level of α-SMA. It is of note that the extracellular matrix molecules, mainly described for breast tumors, have emerged as an important cell regulator, which may affect tumor cell behavior (16,17). The present results revealed that the extracellular matrix protein fibronectin alone was able to upregulate α-SMA expression. The effect of α-SMA upregulation on benign myoepithelial cells as observed in the present *in vitro* model is, however, unclear.

Furthermore, no difference in FGF-2 mRNA expression was detected when the cells were cultured in the tumor-conditioned medium, despite the presence of fibronectin substratum. An upregulation in FGF-2 expression has previously been observed in benign myoepithelial cells when using a squamous cell carcinoma cell line, suggesting that the excessive release of FGF-2 favored malignant cell growth. The same finding was observed when the cells were cultured in fibronectin substratum (10), emphasizing the importance of this extracellular matrix protein in modifying myoepithelial cell function. The tumor media from breast carcinoma and melanoma used in the present study did not influence FGF-2 mRNA regulation. This finding may indicate that there is no correlation between FGF-2 secretion and the quantity of cytokines and growth factors released by these tumors in the microenvironment, which could otherwise promote tumor progression and dissemination (20,21). In this context, using squamous cell carcinoma in the proposed *in vitro* model, the myoepithelial cells appeared to have favored tumor growth via the production of IL-6 and IL-10 stimulated by the malignant cells, in a paracrine way (22). This suggests that the neoplastic benign myoepithelial cells from PA are most likely tumor-dependent, which does not corroborate the finding from previous studies that the myoepithelial cells from PA, albeit transformed, can be used as a surrogate for normal mammary myoepithelial cells (23–25).

The present data resulted in the conclusion that the tumor-conditioned medium obtained from breast ductal adenocarcinoma and melanoma cells did not act on myoepithelial cell differentiation and function, which was revealed by the lack of increase in α-SMA and FGF-2 expression, respectively. Additionally, in the case of the aforementioned malignant tumors, other factors that were not the focus of the present investigation may be playing a role in myoepithelial cell behavior.
